# Rice quality: How is it defined by consumers, industry, food scientists, and geneticists?

**DOI:** 10.1016/j.tifs.2019.07.039

**Published:** 2019-10

**Authors:** Marie Claire Custodio, Rosa Paula Cuevas, Jhoanne Ynion, Alice G. Laborte, Maria Lourdes Velasco, Matty Demont

**Affiliations:** International Rice Research Institute (IRRI), Los Baños, Laguna, Philippines

**Keywords:** Value chain upgrading, Intrinsic attributes, Perception, Sensory evaluation, Gene × environment

## Abstract

**Background:**

Quality is a powerful engine in rice value chain upgrading. However, there is no consensus on how “rice quality” should be defined and measured in the rice sector.

**Scope and approach:**

We adopt a Lancasterian definition of rice quality as a bundle of intrinsic and extrinsic attributes. We then review how rice quality is (i) perceived and defined by consumers and industry stakeholders in rice value chains in Southeast and South Asia; (ii) measured and defined by food technologists; and (iii) predicted through genetics.

**Key findings and conclusions:**

Consumers are heterogeneous with respect to their perceived differentiation of rice quality among regions, countries, cities, and urbanization levels. Premium quality is defined by nutritional benefits, softness and aroma in Southeast Asia, and by the physical appearance of the grains (uniformity, whiteness, slenderness), satiety, and aroma in South Asia. These trends are found to be consistent with industry perceptions and have important implications for regional and national breeding programs in terms of tailoring germplasm to regions and rice varieties to specific local market segments. Because rice is traded internationally, there is a need to standardize definitions of rice quality. However, food technologists have not reached unanimity on quality classes and measurement; routine indicators need to be complemented by descriptive profiles elicited through sensory evaluation panels. Finally, because rice quality is controlled by multiple interacting genes expressed through environmental conditions, predicting grain quality requires associating genetic information with grain quality phenotypes in different environments.

## Highlights

• Consumers perceive rice quality differently among regions, countries, and urbanization levels.
• In Southeast Asia, nutritional benefits, softness and aroma define premium quality.
• In South Asia, physical appearance of grains and satiety define premium quality.
• Current rice quality protocols and classification ranges need to be standardized.
• Rice quality is coded by genes whose expressions are affected by the environment.

## 1 Introduction

Quality is a powerful engine in food value chain (VC) upgrading in developing countries. However, the concept is abstract; for example, in the rice sector, there is currently no uniformly applicable definition of “rice quality” and there is even less unanimity on how it should be measured. Agronomic traits are measured by their ability to increase yields and/or alleviate certain biotic or abiotic stresses of crops; in contrast, measuring quality attributes is not as straightforward because “rice quality” is relative and context-specific (Fitzgerald, McCouch & Hall, 2009). For example, what is considered “low quality” for rural consumers in India may be perceived as “premium quality” by urban consumers in Senegal (Demont, Rutsaert, Ndour, & Verbeke, 2013). Rice quality remains in the eye (and in the mouth) of the beholder—the consumer—and since rice consumption is embedded in a historical, geographical, and socio-cultural context (Cuevas, de Guia, & Demont, 2017; Kaosa-ard & Juliano, 1992), a universal, intercultural construct of “rice quality” is required. Understanding how the market and the industry perceive rice and differentiate it into quality classes could contribute to more efficient, demand-driven, and sustainable rice VCs (Demont, Fiamohe, & Kinkpé, 2017; Demont & Ndour, 2015; My, Demont, Van Loo, de Guia, et al., 2018).

Rice, as a product, is a bundle of characteristics which gives rise to its utility. Through these characteristics, consumer preferences are expressed (Lancaster, 1966). Perception of rice quality may also be judged based on these characteristics or attributes, which could be classified as either *intrinsic* or *extrinsic* (Demont & Ndour, 2015). The former refers to grain quality traits such as color, cleanliness, purity, grain shape and size, uniformity of size and shape, head rice (HR), softness, and aroma while extrinsic attributes include packaging, labeling, and branding. These attributes acquire meaning through the historical, geographical, and socio-cultural context in which rice consumption is embedded. For instance, rice which can be cooked firm and dry is widely preferred by South Asian consumers because the combination of these two attributes characterizes parboiled rice, which is traditionally consumed in the region (International Rice Research Institute, 2016). Another example is the preference for broken rice by Senegalese consumers as a result of long-term importation of cheap broken rice from Asia (Demont et al., 2013).

In this article, we aim to provide an inter-disciplinary review of how rice quality is (i) perceived and defined by consumers and the industry in Asia; (ii) measured and defined in the realm of food science and technology; and (iii) predicted through genetics. We conducted an expert-based literature review by inviting key experts to review the literature on rice quality within their disciplines; i.e., (i) consumer research, (ii) value chain analysis, (iii) food science, and (iv) genetics. Each of the experts relied on their personal expertise, their networks of partners, and their knowledge of the state-of-the-art literature within their disciplines related to the topic of rice quality. Both peer-reviewed literature and IRRI's internal country reports were reviewed and an emphasis was given to the more recent literature. The experts regularly reviewed the sections within the manuscript outside their disciplinary area to ensure that similar quality traits were discussed along the four disciplinary areas to obtain a complete inter-disciplinary Lancasterian overview of rice quality as a bundle of intrinsic and extrinsic attributes. We conclude with a concrete set of recommendations for VC upgrading to rice breeders, food technologists, geneticists, industry stakeholders, and policy makers.

## 2 Rice quality as perceived by consumers and the industry in Asia

To obtain a thorough understanding of how rice quality is perceived and differentiated throughout rice VCs in Asia, we review evidence from multi-country consumer surveys and key informant interviews with industry stakeholders conducted in selected countries in Southeast Asia (SEA) and South Asia (SA) in 2013–2014 (Custodio et al., 2016a, Custodio et al., 2016b, Custodio et al., 2016c, Custodio et al., 2015, Diaz et al., 2014, Velasco, Custodio, Laborte, & Suphanchaimat, 2015, Velasco et al., 2016) The consumer surveys covered 24 cities across eight locations (Philippines, Indonesia, Thailand, Vietnam, Cambodia, Eastern and Southern India, and Bangladesh), and 13 rural districts in Eastern India and in Bangladesh. The surveys were complemented with focus group discussions (FGDs) with farmers and with key informant interviews with industry stakeholders (i.e. traders, millers/processors, exporters, wholesalers, and retailers) operating along the rice VCs serving the urban markets in six out of the eight locations (see supplement S2 for sample size).

### 2.1 Intrinsic quality attributes

#### 2.1.1 Quality perception of consumers in Asia

**Fig. 1:**
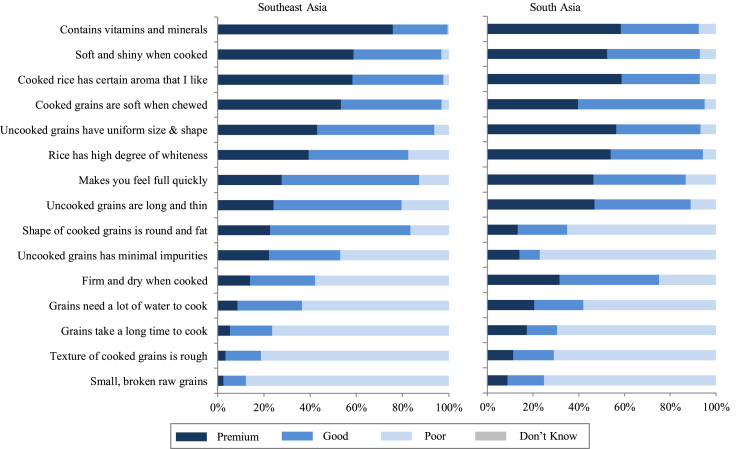
Perception of premium-, good-, and poor-quality rice of urban consumers based on surveys in 24 cities in selected Southeast and South Asian countries in 2013–2014. The perception was based on a single-answer question with 15 pre-defined statements. For each statement, respondents were asked which of the three quality levels they associate it with. *Sample sizes and locations:* Refer to S1 and S2. *Sources:*Custodio et al., 2016a, Custodio et al., 2016b, Custodio et al., 2016c, Custodio et al., 2015, Velasco, Custodio, Laborte, & Suphanchaimat, 2015.

**Fig. 2:**
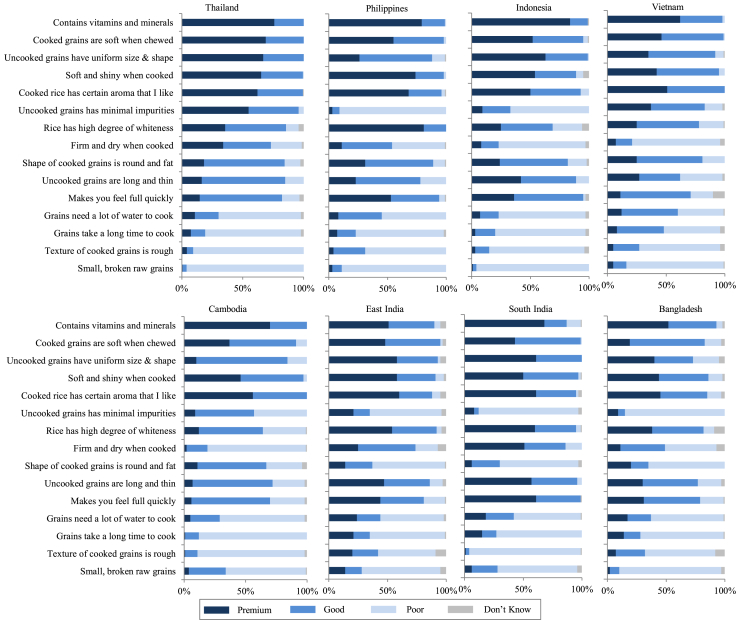
Perception of premium-, good-, and poor-quality rice of urban consumers by country based on surveys in 24 cities in selected Southeast and South Asian countries in 2013–2014. The perception was based on a single-answer question with 15 pre-defined statements. For each statement, respondents were asked which of the three quality levels they associate it with. *Sample sizes and locations:* Refer to S1 and S2. *Sources:*Custodio et al., 2016a, Custodio et al., 2016b, Custodio et al., 2016c, Custodio et al., 2015, Velasco, Custodio, Laborte, & Suphanchaimat, 2015.

**Fig. 3:**
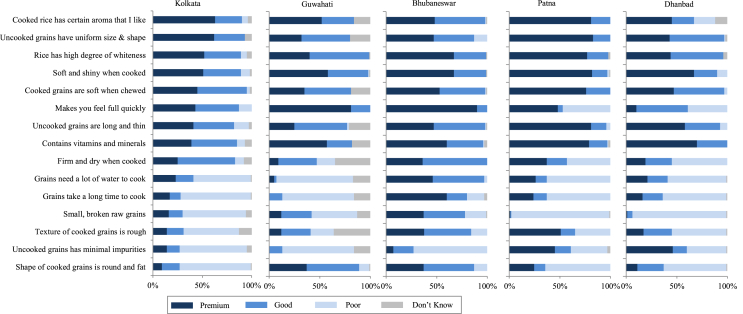
Perception of premium-, good-, and poor-quality rice of urban consumers based on surveys in five cities in Eastern India in 2013–2014. The perception was based on a single-answer question with 15 pre-defined statements. For each statement, respondents were asked which of the three quality levels they associate it with. *Sample sizes:* Kolkata *n* = 201, Guwahati *n* = 150, Bhubaneswar *n* = 150, Patna *n* = 150, Dhanbad *n* = 152. *Sources:*Custodio et al., 2016b, Custodio et al., 2016c, Custodio et al., 2015.

**Fig. 4:**
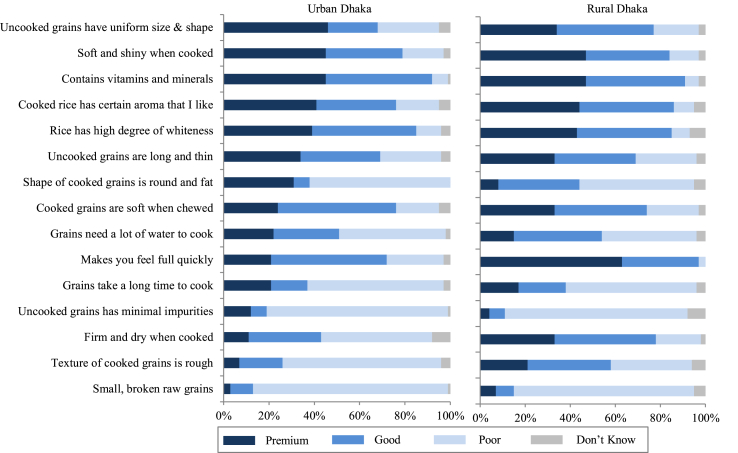
Perception of premium-, good-, and poor-quality rice of consumers in urban and rural districts of Dhaka, Bangladesh based on surveys in 2013–2014. *Notes:* The perception was based on a single-answer question with 15 pre-defined statements. For each statement, respondents were asked which of the three quality levels they associate it with. *Sample sizes:* Urban Dhaka *n* = 296, Rural Dhaka *n* = 296. *Sources:*Custodio et al., 2016a, Custodio et al., 2016b, Custodio et al., 2015.

**Fig. 5:**
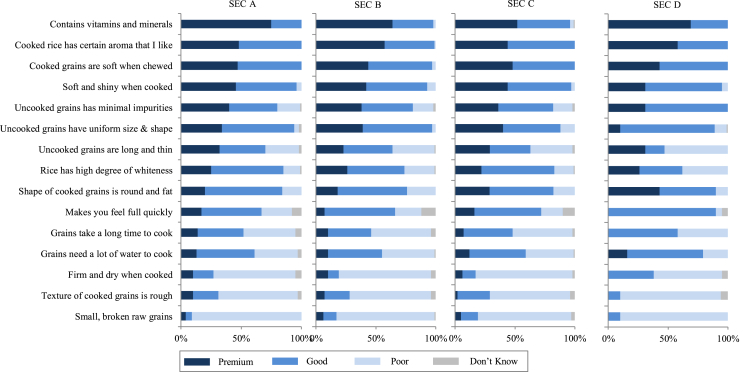
Perception of premium-, good-, and poor-quality rice of urban consumers by socio-economic classes (SECs) based on surveys in Vietnam in 2013. The perception was based on a single-answer question with 15 pre-defined statements. For each statement, respondents were asked which of the three quality levels they associate it with. SEC, as an indicator of a household's (HH) affluence level, is a spectrum wherein a household falls into one of the classes A,B, C, D; SEC A is the most affluent and SEC D is on the other end of the spectrum. In Vietnam, the HH's monthly income is the basis of SEC. *Sample sizes:* SEC A *n* = 50, SEC B *n* = 110, SEC C *n* = 107, SEC D *n* = 32. *Locations:* Refer to S1. *Source*: Custodio, Demont, Laborte, & Ynion, 2016

Rice quality is context-specific and consumers are heterogeneous with respect to how they perceive and differentiate quality. Custodio, Demont, Laborte, and Ynion (2016) and Calingacion, Laborte, Nelson, Resurreccion et al. (2014), for example, illustrate how consumer preferences for grain quality attributes are geographically segmented across SEA and SA. In response to consumer heterogeneity, the rice industry tends to supply a wide range of quality classes, from poor- to good- to high-quality rice, tailored to different market segments. Consumers’ perceptions of rice quality classes were surveyed across SEA and SA; the results are visualized in Fig. 1, Fig. 2, Fig. 3, Fig. 4, Fig. 5. Consumers were asked to classify a set of pre-defined grain quality attributes among three different quality classes; the bar diagrams represent the proportions of consumers who classified the attributes as being representative for “poor”, “good”, or “premium” rice quality. The first general observation that can be made is that consumers do not unanimously perceive these quality classes as being distinct from one another; i.e., there is substantial overlap between classes with respect to the attributes that define them. A single attribute can be perceived as being representative for all three quality classes. The second general observation is that although perceived quality differentiation is heterogeneous among regions (Fig. 1), countries (Fig. 2), cities (Fig. 3), and urbanization levels (Fig. 4), it is remarkably homogeneous among socio-economic classes (SECs) within a country (Fig. 5).

Fig. 1 juxtaposes urban consumers' quality perception in SEA *versus* SA. In SEA, premium-quality rice is perceived to feature nutritional benefits (i.e., contains vitamins and minerals), softness (i.e., soft and shiny, and soft when chewed), and aroma. Although perceived as premium, nutritional benefit is not necessarily a highly preferred attribute by consumers in the region (Custodio et al., 2016b). Since consumers normally eat rice as white polished grains wherein vitamin and mineral content is either minimal or absent (Fitzgerald, Bergman, et al., 2009; IRRI, 2016), the perceived “premium-ness” of nutritional benefit does not seem to translate to preference and consumption. One possible explanation may be limited physical and economic access to healthier rice or that characteristics of available healthier options do not match their preferences. Unlike the nutritional benefit of rice, softness and aroma are preferred characteristics of consumers in SEA (Custodio et al., 2016b; Wangcharoen, Phanchaisri, Daengpok, Phuttawong, Hangsoongnern, &  Phanchaisri, 2016). The perceived characteristics of good-quality rice are uniformity of size and shape, whiteness, long and thin uncooked grains (i.e., long and slender), and round and fat cooked grains (i.e., bold cooked grains). The latter suggests that consumers in SEA, in general, prefer rice that exhibit volume expansion rather than enhanced grain elongation. Good-quality rice is likewise perceived to provide higher satiety (i.e., makes you feel full quickly). Poor-quality rice is perceived to feature impurities, firm and dry texture, rough texture, poor cooking quality, and small broken grains. In SA, perceived quality differentiation by urban consumers is similar to consumers in SEA except for a few attributes. First, what is considered “good-quality” rice by most consumers in SEA (i.e., uniformity of grain size and shape, whiteness, slenderness of uncooked rice grains, and higher satiety) is dominantly perceived as “premium rice” by consumers in SA. On the other hand, although bold cooked grains are associated with good-quality rice in SEA, in SA they are associated dominantly with poor-quality rice. These findings agree with SA consumers’ general preference for slender grains (Custodio et al., 2016b); a shift from preference for medium-shaped grains in the 1980s (Juliano & Villareal, 1993, pp. 12–34). Secondly, while the majority of consumers interviewed in SA consider firm and dry texture a characteristic of either premium- or good-quality rice, most consumers in SEA consider this as an indicator for poor quality. One possible explanation for this is that consumers in SA probably acquired the preference for firm and dry cooked rice grains, such as those with high AC (Graham, 2002), which is a typical quality attribute generated by parboiling of rice, a traditional practice in SA (Custodio et al., 2016a, Custodio et al., 2016b, Custodio et al., 2016c). In both regions, the presence of small broken grains is perceived by consumers as a characteristic of poor-quality rice. In the 1980s, Asian consumers tended to focus on HR (i.e., ratio of whole grains to broken grains) as an important quality characteristic (Damardjati & Oka, 1992; Sriswasdilek, Kongseree, & Attaviriyasook, 1992). Even until the 2000s, in some developing countries, HR was a major determinant of quality grades in urban markets (Rachmat, Thahir, & Gummert, 2006). However, due to progressive upgrading of milling technology and post-milling operations in Asia (Barker, Herdt, & Rose, 1985, pp. 172–184), high HR has become a minimum quality standard and attention has now shifted to grain homogeneity (uniform grain size and shape) as a criterion for premium- or good-quality rice.

When we zoom in at country level, more differences in perceptions appear (Fig. 2). In Thailand, a lead exporter in the region, quality perceptions of urban consumers largely reflect the regional average for SEA (Fig. 1). A noticeable difference, though, is that Thai consumers perceive rice with firm and dry texture as a characteristic of good quality, whereas this characteristic is mostly attributed to poor-quality rice in other SEA countries (except in the Philippines). This finding calls for further investigation because previous studies (e.g., Calingacion et al., 2014) indicate general preference of Thai consumers for sticky texture (0–2% amylose content, AC) to quite soft and sticky (low AC; 10–19%) in the Northern and Northeastern regions and for firmer and less sticky texture (intermediate AC; 20–25%) in the Central Plains (AC ranges from Kumar & Khush, 1986; descriptions from Tuaño, Regalado, & Juliano, 2016). Another interesting finding in Thailand is the perception of long and slender grains and whiteness as characteristics of good-quality rice. Although historically, these are preferred characteristics of rice (Unnevehr, 1986; Juliano & Villareal, 1993, pp. 12–34; Calingacion et al., 2014; Custodio et al., 2016b), another recent study found that Thai consumers consider aroma, taste, and texture more important than color (e.g., whiteness) and appearance (e.g., size and shape) (Wangcharoen et al., 2016). Thai consumers may have now considered color and appearance as minimum requirements and hence are no longer considered as characteristics exclusively of premium-quality rice. In SEA, the perception of premium-quality rice of Thai consumers is prominently reflected in the Philippines and in Indonesia and to a lesser extent in Vietnam and in Cambodia (Fig. 2). This finding suggests that Thailand's exports may have also influenced rice quality perception of importing countries, similarly with their influence on preferences (Custodio et al., 2016b). Similar to Thai consumers, consumers in Vietnam and in Cambodia discriminate nutritional benefit and aroma of rice as characteristics of premium-quality rice. We observed that quality perception of consumers in these two countries is similar in the same manner that rice preferences are shared (Custodio et al., 2016b). In Eastern and Southern India, the perceived attributes of premium-quality rice pertain to nutritional benefit, to uniformity of size and shape, and to aroma. The majority of consumers surveyed in Eastern and Southern India perceive whiteness as a characteristic of premium-quality rice. This may seem contradictory because parboiled rice is widely consumed in SA and the process of parboiling traditionally makes rice yellowish. However, modern processing technology has made it possible for parboiled rice to be more white/less yellow (Custodio et al., 2016a, Custodio et al., 2016c) which could have made white rice available to consumers, even in parboiled form. Additionally, whiteness is a preferred rice characteristic of urban consumers in these locations (Custodio et al., 2016b). The perception of poor-quality rice seems to be similar among consumers across the eight locations surveyed: small and broken grains, rough texture, long cooking time, and too much water requirement for cooking.

Even within a country, quality perception is far from homogenous and can vary from city to city. We illustrate this through the case of Eastern India (Fig. 3). Urban consumers in some cities seem to be less demanding than consumers in other cities. For example, many of the attributes that are associated with premium- and good-quality rice in Kolkata are mainly considered premium attributes in Patna. Secondly, although presence of small and broken grains, high water requirement for cooking, and long cooking time are perceived by consumers in most cities as characteristics of poor-quality rice, in Bhubaneswar these are mostly perceived as characteristics of either premium- or good-quality rice. Finally, although minimal impurities is a minimum requirement in most cities, it is still considered a criterion for premium quality in Patna and Dhanbad. Heterogeneity in rice quality perception is also evident between urban and rural consumers, illustrated through the case of Dhaka, Bangladesh (Fig. 4). Firm and dry texture and higher satiety, for example, are perceived more positively (premium or good quality) by rural consumers than by urban consumers. One of the reasons why rural populations tend to prefer parboiled rice is its higher satiating power, which helps rural workers maintain their energy levels for agricultural work. Remarkably, perceived quality differentiation is fairly similar across SECs, illustrated through the case of urban consumers in Vietnam (Fig. 5). The fact that this is similar in all countries (Custodio, Paguirigan, Laborte, Ynion, & Demont, 2015; Custodio et al., 2016a, Custodio et al., 2016b, Custodio et al., 2016c, Velasco, Custodio, Laborte, & Suphanchaimat, 2015) suggests that even the less affluent consumers seem to have a well-defined perception of the different quality classes even if low-income consumers, particularly in rural areas, do not seem to have economic power to express their preferences (Cuevas, Pede, McKinley, Velarde, & Demont, 2016).

#### 2.1.2 Quality perception of rice farmers and industry stakeholders in Asia

To assess transmission of perceived quality differentiation along rice VCs in SEA and SA, we summarize some evidence from key informant interviews with VC actors and FGDs with farmers (Table 1; Custodio et al., 2016a; Custodio et al., 2016c; Diaz et al., 2014; Velasco et al., 2015; Velasco et al., 2016). The first general observation that can be made is that farmers’ perceptions of quality classes are fairly consistent with other VC actors. Hence, in the succeeding discussion, we refer to VC actors to include farmers and other actors in the VC. Within each region, industry perceptions are fairly consistent with consumer perceptions. In SEA, premium-quality rice is characterized by soft texture, long and slender grains, whiteness, translucency, aroma, and high HR (i.e., minimal or no broken grains). Like consumers (Fig. 2), VC actors in Thailand and Vietnam perceive rice with nutritional benefits as premium quality.

**Table 1:** Perception on premium-, good-, and low-quality rice of value chain actors in selected locations in South and Southeast Asia in 2013–2014.

Location	Attribute	Premium quality	Good/Medium quality	Low quality
Thailand	*Texture*	Soft^d^; Slightly sticky (in the Northeast region)	Soft; Becomes hard when cooled; Slightly sticky	Becomes hard when cooled
	*Size and shape*	Long^d^	Long^d^	Short^d^
	*Color*	White; Translucent; Not chalky	White; Translucent but less than premium; Not chalky^d^	Chalky^d^
	*Aroma*	With fragrance^d^	With fragrance	No fragrance
	*Purity*	No impurities^a^		
	*Homogeneity*	High HR; Uniform size, shape, color	With variations in size & shape; With 5% broken grains and color variation	Higher % of broken grains; With variations in size, shape, color
	*Others*	Shiny; Good cooking quality; High nutritional value^d^; High volume expansion	High volume expansion	Poor cooking quality; Not tasty
Philippines	*Texture*	Soft^d^	Moderately soft after cooking but hard when cooled	Becomes hard when cooled
	*Size and shape*	Long^d^; Slender	Long^d^;Slender	
	*Color*	White^d^		Yellowish; Discolored^d^;
	*Aroma*	With fragrance	No fragrance	With bad smell^d^
	*Purity*			
	*Homogeneity*	High HR^d^	With 5–15% broken grains; High HR^d^	With 25% broken grains^d^
	*Others*	High volume expansion; Tasty^d^		Not shiny
Vietnam	*Texture*	Soft^d^; Sticky^d^		Non-sticky; Becomes hard when cooled^d^; Dry^d^
	*Size and shape*	Slender	Long	Short^d^
	*Color*	White; Translucent		Yellowish; Chalky^d^
	*Aroma*	With fragrance^d^		
	*Purity*			With impurities^a^
	*Homogeneity*	High HR	With 10–15% broken grains	High % of broken grains; With variations in size, shape and color
	*Others*	High nutritional value^d^; Tasty; Low volume expansion	Sweet; Medium volume expansion	Not sweet; High volume expansion^d^
Cambodia	*Texture*	Soft; soft and sticky	Soft but not as soft as premium; Soft and sticky; Not too sticky	Becomes hard when cooled
	*Size and shape*	Slender; Long	Slender; Medium size	
	*Color*	Translucent	Less chalky grains	Chalky
	*Aroma*	With fragrance	With fragrance	No fragrance
	*Purity*	No impurities^a^		
	*Homogeneity*	High HR	High HR; Uniform size, shape, color	High % of broken grains
	*Others*	Shiny; Tasty	Shiny; Tasty; Longevity^b^	Not shiny; Not tasty
Eastern India	*Texture*	Soft^d^; Non-sticky^d^		Sticky^d^; Very soft
	*Size and shape*	Long; Slender^d^; Very fine to fine^c^^,^^d^	Less slender than premium; Fine^c^	Short^d^; bold^d^
	*Color*	White		Discolored
	*Aroma*	With fragrance	With fragrance; Less aromatic than premium	No fragrance
	*Purity*	No impurities^a^	No impurities^a^	With impurities^a^
	*Homogeneity*	High HR; Uniform size, shape, color^d^	High HR; With 5% broken grains, color variation, admixture	High % of broken grains; With variations in size, shape and color^d^
	*Others*	Shiny; Less cooking time^d^; Very tasty^d^; Easy to digest	Polished but not very shiny; Tasty	Long cooking time; Not tasty^d^
Bangladesh	*Texture*	Non-sticky	Non-sticky	Sticky; Soft/becomes too soft if cooked rice is soaked in water overnight
	*Size and shape*	Long^d^; Slender; Very fine to fine^c^^,^^d^	Fine to medium fine^c^^,^^d^; Medium size	Coarse^c^^,^^d^; Bold
	*Color*	White	White but not as white as premium; white even if parboiled	Not very white
	*Aroma*	With fragrance^d^	No bad smell	With bad smell
	*Purity*		With 5% impurities^a^^,^^d^	With impurities^a^
	*Homogeneity*		With 5% broken grains	Higher % of broken grains
	*Others*	Tasty^d^; Longevity^b^	Tasty^d^; Longevity^b^	Not tasty^d^

*Notes:* The data are based on key informant interviews with industry stakeholders and focus group discussions with farmers. *Sample sizes and locations:* Refer to S1 and S2. *Sources:*Custodio et al., 2016a, Custodio et al., 2016c, Diaz et al., 2014, Velasco, Custodio, Laborte, & Suphanchaimat, 2015, Velasco et al., 2016.

a Impurities refer to black grains, stones, or husks.

b Longevity refers to cooked rice which does not easily spoil.

c There are different classifications of grains in South Asia. Very fine to fine grains refer to extra-long and long slender grains, medium fine grains refer to medium slender grains, coarse grains refer to short bold grains.

d Refer to attribute which was also mentioned by farmers in the FGDs.

Good-quality rice is perceived to have soft texture—but not as soft as premium—and becomes hard when cooled and has a small amount of broken grains. Similar with consumers, VC actors have different perceptions towards aroma as a quality attribute. For instance, VC actors in Thailand and in Cambodia consider aroma as a characteristic of good- and premium-quality rice but for VC actors in the Philippines, good-quality rice can be non-aromatic. Grain size and shape requirements for good-quality rice also appear to differ across the countries surveyed. In Cambodia, rice with medium-sized grains is considered good quality while in Vietnam, the Philippines, and Thailand, good-quality rice is long and slender. The acceptable level of broken grains varies across the different countries: VC actors in Thailand seem to have more stringent standards because the acceptable level of broken grains for good-quality rice is 5%; in contrast, VC actors in the Philippines and Vietnam accept up to 15% broken grains.

Low-quality rice is similarly perceived by the VC actors in SEA as rice that hardens upon cooling and has low grain homogeneity. In Vietnam and in the Philippines, yellowish grains were also deemed by VC actors as low-quality rice. High volume expansion is perceived as characteristic of low-quality rice by VC actors in Vietnam. However, this characteristic is perceived by VC actors in Thailand and the Philippines as a property of premium-quality rice. VC actors in Thailand seem to be aware of consumer preference, in all SECs, towards high volume expansion, a feature of aged rice (Custodio et al., 2016b; Velasco et al., 2015). In the Philippines, high volume expansion is preferred by consumers in the middle and in the lower SECs and is seen as an economical way to feed the family (Custodio et al., 2016b). Perhaps this is a reason why rice varieties with high volume expansion are considered as premium-grade rice.

Industry stakeholders interviewed in SA have fairly similar perceptions of premium-, good-, and poor-quality rice as consumers in the region. The perceived characteristics of premium-quality rice are non-sticky texture, extra-long to long slender grains (i.e., very fine to fine grains), and fragrance (Table 1). Good-quality rice is generally perceived to have long slender or medium slender grains (i.e., fine or medium fine grains), white and polished—but not as much as premium—and high HR (i.e., with 5% broken grains). VC actors in Eastern India appeared to be more demanding than those in Bangladesh. For instance, VC actors in Eastern India consider aroma as a characteristic of good- and premium-quality rice; for VC actors in Bangladesh, however, as long as the rice has no unpleasant smell, it is considered of good quality. Low-quality rice, on the other hand, was associated with no aroma in Eastern India and with unpleasant smell in Bangladesh. Also, VC actors in Eastern India deem that good-quality rice should be clean but VC actors in Bangladesh allow the presence of 5% impurities for good-quality rice. In both locations, low-quality rice is perceived to have sticky texture and very soft texture, short and bold grains (i.e., coarse) and high amount of broken grains and impurities (i.e., black grains, stones, husks).

### 2.2 Extrinsic quality cues

The definition and perception of rice quality can be further reinforced through extrinsic quality cues such as labeling, branding, and packaging. Extrinsic quality cues are powerful drivers for building value of a rice product, for reducing quality uncertainty, and for forming quality expectations (Demont et al., 2013; Demont & Ndour, 2015; My et al., 2018) through product information. Whether rice is sold in loose or in packaged format, product information can be conveyed in several ways such as in-store merchandise, communication with the retailers, and in packaging.

Reardon, Chen, Minten & Adriano (2014) observed a shift in marketing rice from loose to packaged rice in Asia. Loose means rice bought in small portions and the seller packs accordingly (sealed or placed in unsealed bag) while packaged means the bag is sealed or pre-packed. Our findings support this transformation happening in some locations. Among consumers surveyed in the different cities in Asia, we found that the purchase of packaged rice is more prominent in Thailand, Cambodia, South India, and Bangladesh while rice is mostly purchased in loose format in the Philippines, Vietnam, and Eastern India (Table 2). Majority of consumers interviewed in the Philippines purchase rice from traditional channels particularly from neighborhood stores or “sari-sari” stores where a variety of products are sold by piece or in small portions. In Vietnam, there are more consumers who purchase rice from rice wholesalers-retailers (i.e., sellers who specialize in selling rice either in wholesale or retail) who they can request to “custom-pack” small portions and even to mix different kinds of rice.

**Table 2:** Rice package format and purchase channels of urban consumers based on consumer surveys in 24 cities in selected Southeast and South Asian countries in 2013–2014 (% of respondents per location).

	Southeast Asia					South Asia		
	Thailand	Philippines	Indonesia	Vietnam	Cambodia	East India	South India	Bangladesh
**Package format**								
Loose rice only	34	82	49	84	45	80	18	32
Packaged rice only	62	18	44	16	53	17	70	62
Loose and packaged	5	0	7	0	2	3	12	6

**Purchase channel**								
Traditional retailers only	57	93	98	98	100	98	96	98
Modern retailers only	37	5	2	1	0	2	3	2
Both traditional and modern	6	2	1	0	0	0	2	0

*Notes:* Descriptions of loose and packaged rice were read out to respondents as follows: “Loose rice means you can buy in small portions such as kilogram or even half a kilo and the retailer packs accordingly. Packaged rice means the bag is sealed or pre-packed with or without name of variety or label or origin indicated in the bag”. Traditional retailers refer to wet/fresh market or market stalls, neighborhood variety stores, grocery stores, mom & pop shops, and rice dealers. Modern retailers refer to supermarkets, hypermarkets, and convenience stores or mini markets. *Sample sizes and locations:* Refer to S1 and S2. *Sources:*Custodio et al., 2016a, Custodio et al., 2016b, Custodio et al., 2016c, Custodio et al., 2015, Velasco, Custodio, Laborte, & Suphanchaimat, 2015

Our observations on *packaging* and *information* on the package confirm the role of extrinsic quality cues in *building value* for the product. Rice is mostly bought in traditional channels in the countries surveyed (with the exception of Thailand), even where purchase of packaged rice is prominent (Table 2). The packaged rice bought by consumers in Vietnam, in Cambodia, and in Bangladesh has information on the variety name, label, or origin but the details were not elaborated in our survey. This information was not collected in the other countries but we hypothesize that the same may be true, especially in Thailand where branding seems to be well advanced (Velasco et al., 2015). In India, it was reported by Reardon et al. (2014) that mill information, type of rice, and, sometimes, trader information are typically featured on the rice package. In Vietnam, My et al. (2018) found that urban consumers were willing to pay price premiums for rice that was labeled and certified as being sustainably produced and even more so when supplementary information on certification and traceability was provided.

Consumers' awareness of different rice types through their *labels* likewise confirms that extrinsic quality cues are powerful drivers in *building value for the product* (e.g., equity of a specific variety) and *forming quality expectations* (e.g., intrinsic attributes of a specific variety). In the context of branding, “equity” is related to the degree of brand name recognition, to perceived quality, and to mental and emotional associations. Rice is sold and marketed to consumers through different labels such as variety name, trade name or a generic term adopted by traders, a brand name which identifies its sellers (e.g., company) to differentiate their product from that of competitors, and rice type. We attempted to measure the saliency of these rice labels by looking at the average proportion of consumers being aware of these classifications. In Eastern India, we classified the rice labels as trade/brand name, rice type, and variety name (Table 3). Variety names include official release names, alternative names, and local variety names. We classified Basmati separately to include brand/trade names for Basmati and any Basmati variety. We observed that awareness is more diverse among urban consumers in Patna and Dhanbad. Brand/trade names are salient among urban consumers in Guwahati. Both brand/trade names and variety names are salient among consumers in Bhubaneswar.

**Table 3:** Average proportion of consumers’ awareness of rice brands, types and varieties in selected cities and rural districts in Eastern India based on surveys conducted in 2013–2014 (% of respondents per city).

	Cities					Rural districts	
Rice label	Kolkata	Guwahati	Bhubaneswar	Patna	Dhanbad	Rural West Bengal	Rural Odisha
Brand/trade name	31	45	43	25	31	30	33
Basmati	25	13	12	24	21	19	14
Rice type	16	14	3	27	25	6	2
Variety name	25	27	42	21	23	44	51
Others	1	1	0	3	1	1	0
None/Don't know	3	1	0	0	0	0	0

*Notes:* Per respondent, we first list all brand, type and variety responses and then calculate the proportion of each category among all responses. Trade names are generic terms adopted by traders for particular rice. Brand names identify the sellers (e.g. company) to differentiate their product from competitors. For simplicity, we treated brand and trade name under one classification. Rice types refer to parboiled rice, raw or non-parboiled rice, and “arwa” (slightly polished non-parboiled rice). We classified Basmati separately to include brand/trade names for Basmati and any Basmati variety. Variety names include official release names, alternative names and local variety names. For each respondent, the percent awareness of each of the rice classification was computed to determine the average proportion. *Sample sizes: Refer to S2. Sources:*Custodio et al., 2016b, Custodio et al., 2016c, Custodio et al., 2015

The awareness of the trade name “Barpetiya” is an example of *quality expectation* formed on the basis of geographic origin (i.e., provenance). “Barpetiya” is widely known to originate from Barpeta District in Assam State and has high awareness among consumers in Guwahati which is located in Assam State (Custodio et al., 2016c). It has two specific types: “Aijong” and “Joha” Barpetiya Aijong” is known to be non-aromatic, to have medium fine grains, and to be neither so sticky nor soft. “Barpetiya Joha” is known to be aromatic, to have short grains, and to be somewhat sticky. Consumer awareness of Barpetiya indicates that provenance of the product is becoming increasingly part of consumer quality perception and hence, implies perception on its authenticity and sustainability.

The popularity of Mahsuri variety in Bhubaneswar illustrates the role of *labels* in *building product value*. Mahsuri variety has high awareness levels among consumers in Bhubaneswar (Custodio et al., 2016c). However, the original Mahsuri variety, which was released in the 1960s, is no longer grown by farmers but the variety name is still very popular. Traders may be labeling other varieties with similar grain quality characteristics as Mahsuri, which suggests that Mahsuri has some level of equity among traders and consumers. This is also the case for Dinorado rice, a type of rice preferred by consumers in the Philippines.

The case of *branding and packaging* in Thailand further reinforces the role of extrinsic quality cues in *building value,* *in* *reducing quality uncertainty* and *in* *forming quality expectations*. Across all the eight locations, it is only in Thailand where modern retailing seems to be evident (Table 2). Nearly half of urban consumers interviewed in Thailand purchase rice from modern channels such as supermarkets/hypermarkets. This was prominently observed in Greater Bangkok and among consumers in the upper socio-economic class (SEC AB; Velasco et al., 2015). This finding supports the structural transformation in the retail segment happening in Asia (Reardon et al., 2014) characterized by increased penetration of supermarkets in more developed cities. Branding and packaging likewise seem to be more advanced in Thailand (Table 2). Urban Thai consumers usually purchase branded-packaged rice (e.g., Royal Umbrella, Mah Boon Krong/MBK, Benjarong). Branded rice is perceived to be of better quality as modern retailers consider these to have undergone standard quality checks and better milling and packaging processes (Velasco et al., 2015).

In summary, rice quality is reinforced through the different extrinsic quality cues. Trade names, including variety names used as generic terms, are used to form quality expectations (e.g., the case of “Barpetiya” in Assam State) and to reduce quality uncertainty (e.g., the example of Mahsuri-labeled rice in Bhubaneswar and Dinorado-labeled rice in the Philippines). Prominent rice brands in Thailand reinforce product value through differentiation and by ensuring quality standards. The presence of modern retailing in Thailand also reinforces quality standards imposed on products. The trend in labeling rice to highlight its nutritional content/benefit (e.g., low glycemic index) to consumers in SEA and SA is an area for future research to further the role of extrinsic quality cues in quality perception and more specifically, as a nutrition cue for consumers. Though seen in modern retail stores in more developed countries such as Thailand, labeling is still unregulated in Asia unlike in the developed economies in Europe, the United States of America, and Australia which impose stringent measurements and standards.

## 3 Rice quality as measured and defined by food technologists

Rice quality is context-specific and judgments are based on a hierarchy of values of consumers. However, despite the subjectivity of “quality” as a social construct, several of the intrinsic attributes that people use to judge the quality of rice have indicators that can be quantitatively measured. From a food technology standpoint, rice grain quality is defined by the different combinations of these quantitative indicators. Generally, these metrics can be classified as addressing raw-grain quality (inclusive of appearance and milling quality), cooking and eating quality, or nutritional quality (Kaul, 1970). Some of these classification systems are not yet unified, which can lead to potential impacts in international rice trade and varietal improvement programs. Many of the attributes that distinguish high- from low-quality rice, on the other hand, have no equivalent quantitative indicators; this leads to ambiguity in how rice quality is further defined by food technologists. Hence, here, we discuss in-depth indicators of intrinsic grain quality attributes that are quantifiable and review the knowledge gaps that keep other attributes ambiguous.

### 3.1 Raw-grain quality indicators

Consumers, particularly those who buy loose rice, typically judge rice varieties and make purchase decisions based on their first impressions on the characteristics of the raw grain. Thus, raw-grain quality is a crucial component on the salability of rice varieties in their target markets. These raw-grain attributes mostly deal with the visual properties of the grains, some of which can be quantified. Some of these attributes may not be top-of-mind for consumers (i.e., these attributes are not mentioned unless participants are prompted in surveys) but are important quality indicators because non-compliance to consumer requirements lead to non-purchase. There are also attributes which have different terminology between consumers and food technologists. Hence, in this section of the paper, the attributes evaluated by food technologists may be termed differently from those surveyed from consumers or have been excluded from the surveys.

*Grain size and shape* are strong criteria for purchasing. Breeders normally consider these attributes first in varietal improvement programs (Adair, Beachell, Jodon et al., 1966). Because grain length is more variable than grain width (Anacleto, Cuevas, Jimenez, Llorente, Nissila, et al., 2015), the size is defined, mainly, by grain length. Grain shape, on the other hand, is defined as the ratio of grain length to grain width (Graham, 2002). These values are then used to classify rice into quality classes. However, it appears that classification systems based on grain size and shape feature different ranges of grain lengths and length/width ratios (Table 4); hence, there is no universal definition of the different grain sizes. The system followed by the United Kingdom (HM Revenue & Customs, 2015) indicates that grains with lengths greater than 6.0 mm are classified as long-grain but it does not include the extra-long-grain class reported by Belsnio (1992). Khush, Paule, & de la Cruz, 1979 and Adair et al. (1966) likewise had an extra-long-grain category; however, in their classification system, extra-long grains according to Belsnio (1992) can still be classified as long-grain. Medium- and long-grain are also defined differently by these classification systems. On the other hand, these four reported classification systems seem to agree that grains with lengths approaching 5.0 mm can be considered short. The only exception among these classification systems (Table 4) is the one published by the Codex Alimentarius Commission (1995, pp. 1–6). In this system, grains that can be considered of medium and of long lengths in the other systems may be classified as short grains. In contrast, all five classification systems seem to agree about the ranges for grain shape. Dela Cruz and Khush (2002), on the other hand, published a score scale for visual classification (Table 4). Clearly, there is a need to standardize definitions that can be used globally, especially since rice is traded internationally.

**Table 4:** Comparison of milled rice grain quality classes based on grain size and shape (length/width ratio).

Reference	Grain length (mm) classifications				Grain shape (length/width ratio)		
	Extra long	Long	Medium	Short	Long/Slender	Medium/Bold	Short/Bold/Round
Khush, Paule, & de la Cruz, 1979 Adair et al. (1966)	>7.50	6.61–7.50	5.51–6.60	<5.50	>3.00	2.01–3.00	1.01–2.00
Codex Alimentarius Commission, 1995^a^		≥6.6	6.2–6.5	<6.2	≥3.0	2.0–2.9	<1.9
Belsnio, 1992^b^	≥7.0	6.0–6.9	5.0–5.9	<5.0	>3.0	2.0–2.9	<2.0
HM Revenue & Customs, 2015		>6.0	5.3–5.9	≤5.2	≥3.0	2.0–2.9	<2.0
Dela Cruz & Khush, 2002^c^	1	3	5	7	1	5	9

a Length/width ratios were classified as long, medium, and short.

b Length/width ratios were classified as slender, bold, and round.

c Score scale for visual classification for grain lengths and length/width ratios.

Whiteness is an ambiguous quality indicator elicited from consumers because it may refer to two different grain characteristics. Typically, rice grains are called “white” by consumers when the grains are translucent; also, consumers describe well-milled rice as “white” (i.e., no bran left on the milled grain) (e.g., Fig. 1). The former refers to the degree of chalkiness of the rice grains while the latter alludes to the degree of milling of rice. Chalkiness has an effect on HR recovery, another indicator of milling quality (Raju, Manjunath, & Srinivas, 1991). Because of their economic importance, significant investments have been put in improving these attributes of milled grains.

*Chalkiness* affects the appearance of the grain as it refers to the degree of opacity of the endosperm, whether this occurs in the dorsal side (white back), in the ventral side (white belly), or at the center (white core) of the grain (Ashida, Iida, & Yasui, 2009). Chalkiness is attributed to the presence of intergranular air spaces in the endosperm, which cause light to scatter, and therefore opacity. The opacity of chalky grains is different from that of waxy grains because light is diffused by micropores and hollows in waxy rice, rather than by airspaces between starch granules (Ashida et al., 2009); these micropores appear to not have an effect on the mechanical strength of the waxy grain. Methods for quantifying chalk and classifying rice based on chalkiness have been extensively reviewed. Classifying grains based on a visual assessment of the chalky proportion of the grain is widely used (e.g., Gu, Chen, Chen, Wang et al., 2015). The advent of image analysis capacity has improved the objectivity in classification of rice grains and has allowed for an increasingly quantitative measurement of the proportion of chalky grains (e.g., Yoshioka, Iwata, Tabata, Ninomiya, & Ohsawa, 2007). However, despite improvements in measuring chalk, there is no standard method in classifying grains into different quality categories. For instance, grains can be classified into three groups based on the rating scale: 1 (<10% chalkiness), 5 (10–20% chalkiness), and 9 (>20% chalkiness) (Graham, 2002). Alternatively, grains could be classified into three categories based on grain width and its association with the presence of the white belly (Raju et al., 1991), again through visual inspection. On the other hand, Yoshioka, Iwata, Tabata, Ninomiya, & Ohsawa, 2007 proposed seven classes for chalkiness: perfect rice, white-based, white-back, white-back and white-based, white-belly, white-core, and milky-white. Another approach is to only cite the actual values obtained from imaging analyses. For instance, a study involving 16,860 rice samples showed that chalkiness exhibited a non-normal distribution with a strongly positive skew; the distribution indicated that the majority of the samples tended to have 0–15% chalkiness (Anacleto et al., 2015). The unimodal nature of the distribution further suggests that chalkiness can be seen as a continuum rather than a set of distinct classes.

Because of its effects on visual appearance and HR (which are both strongly linked with consumer preferences, Fig. 2), rice grain chalkiness has implications on the selling price of rice. In the Philippines for instance, it was determined that there is a price penalty for increased chalkiness of loose rice sold in various retail stores (Tuaño, Regalado, & Juliano, 2016).

*Head rice (HR)* pertains to the proportion of milled rice grains that are whole (whose lengths are greater than 75% of the lengths of the unbroken grain) vis-à-vis paddy grain by weight (Graham, 2002). It is one of the most economically important attributes of rice; it has been reported that decreasing proportions of HR is associated with decreasing market value of milled rice (Demont et al., 2017; Demont & Ndour, 2015; Cuevas et al., 2016; Siebenmorgen, Counce, & Wilson, 2011. Because HR is calculated from paddy grain, the theoretical maximum HR that can be obtained is around 70% (Siebenmorgen et al., 2011). There are many factors that could affect HR; hence, in the real world, the maximum HR that can be attained may be 55–60%. Aside from chalkiness, fissures in kernels are associated with reduced HR because these fissures cause mechanical failures within the kernels. These fissures can occur both during and after milling, and are reportedly associated with high temperature gradients in the grain between milling and cooling steps (Pan, Khir, & Thompson, 2013) and with the temperature and the relative humidity of the air while rice is being milled (Siebenmorgen, Saleh, & Bautista, 2009). HR is also reportedly associated with the proportion of strong kernels (those that can withstand 20N of bending force) rather than with the average breaking force of a sample. To reduce the incidence of HR through the reduction of grain fissures, scientists have devised intermittent drying and tempering cycles that can reduce moisture gradients inside the rice grains (Schluterman & Siebenmorgen, 2007). The soak stress test was devised to screen rice varieties for fissuring, and therefore, for HR as well. Imaging techniques, alternatively, can be used to screen for fissuring in paddy or milled grain in a non-destructive fashion. Quantification of HR is typically conducted to screen advanced lines in breeding programs. In classifying rice sold in the market, however, the values expressed are typically based on the proportion of broken grain to milled rice (Section 2.1). Premium-grade rice typically contains up to 5% broken grain while the Grade No. 4 contains around 24% broken grain (National Food Authority, 2013). The differences in the ways of expressing the proportion of head rice may cause confusion, particularly for quality inspectors who evaluate different batches of rice and for breeders who need to set realistic targets for their varietal development pipelines.

### 3.2 Cooking and eating quality indicators

*Amylose content* (AC) is considered to be a major predictor of rice eating quality as it has been associated with mechanical textural attributes such as hardness and stickiness (e.g., H. Li, Prakash, Nicholson, Fitzgerald, & Gilbert, 2016) and it is relatively simple to measure. Methods have been devised to measure AC: based on molecular size distributions, differential scanning calorimetry, precipitation with concanavalin A (reviewed in Kaufman, Wilson, Bean, Herald, & Shi, 2015) and spectroscopy (e.g., Sohn, Himmelsbach, & Barton, 2004). Association of the *Waxy* gene with AC has allowed scientists to utilize known alleles of these genes to predict AC class as well (reviewed in Biselli, Cavalluzzo, Perrini, Gianinetti et al., 2014). The assay of choice for cereal chemists is iodine colorimetry, which depends upon the intensity of the color reaction of the resulting iodine-amylose complex. Efforts to improve this colorimetric approach focused on (i) increasing throughput and ease of measurement by using autosamplers (Juliano, 1992), color charts (Avaro, Tong, & Yoshida, 2009), and 96-well plates (Kaufman et al., 2015); and on (ii) reducing the interaction between iodine and amylopectin, the highly branched homoglucan polymer that is also present in rice starch (Juliano et al., 2012). In attempts to avoid issues related to varying results caused by variations in methods, scientists have conducted international efforts (Fitzgerald et al., 2009) to devise a standard method for measuring AC. These efforts have resulted eventually in the assays published by the ISO (International Organization for Standardization, 2015b, International Organization for Standardization, 2015a). However, the standard methods do not seem to be followed universally yet. Rice can be classified into three to five distinct AC classes, whose ranges have been arbitrarily set, as can be noted based on the differences in ranges per reference (Table 5). It is interesting to note that the range of high AC is constant at >25% across the different reports. However, the ranges of the other AC classes among these reports tend to differ. There are also suggestions to further differentiate low, intermediate, and high AC classes into subgroups (Cagampang, Perez, & Juliano, 1973; Coffman & Juliano, 1987; Juliano, 1992; Calingacion et al., 2014). It is not known if such further differentiation is indeed needed until the relationships of AC with textural attributes, within AC classes, is better understood. Also, there is an apparent misalignment of how food scientists define the different AC classes and how commercial actors define these. From a commercial standpoint, AC classes are low, intermediate/medium, and high (Suwannaporn, Pitiphunpong, & Champangern, 2007), with varieties in very low AC and waxy classes being grouped together with low-AC varieties. Different regions have different AC requirements based on consumer preference (Calingacion et al., 2014) and AC has a detrimental effect on market price of rice (Unnevehr, Juliano, & Perez, 1985), implying that different laboratories releasing different AC values and classifying rice using different systems (Table 5) can lead to potential issues in rice trading (e.g., rejection or acceptance of rice based on AC and pricing issues). Some of the challenges of relying solely on AC are the variation of results one can get from different lots of the same variety (Cagampang et al., 1973; Tuaño, Umemoto, Aoki, Nakamura et al., 2011) and whether there are differences in eating quality for varieties with AC along the endpoints of the AC class ranges (Table 5).

**Table 5:** Comparison of milled rice grain quality classes based on apparent amylose content (AC).

Reference	Apparent amylose content ranges (%)				
	Waxy/Glutinous	Very Low AC	Low AC	Intermediate AC	High AC
Tuaño et al. (2011)	0–2		12–18	18–25	>25
Kumar and Khush (1986)	0–2	3–9	10–19	20–25	>25
Cagampang et al. (1973)	1–2		12–20	20–25	Moderate: 25–27 High: >27
Calingacion et al. (2014)			A: 11–15 B: 16–19	A: 20–22 B: 23–24	A: 25–27 B: >27
Juliano (1992)	0–5	5–12	12–20	20–25	25–33
Suwannaporn et al. (2007)			<20	21–25	26–33
Coffman and Juliano (1987)	1–2		12–19	20–24	25–32
Khush, Paule, & de la Cruz, 1979			10–23	20–25	25–30

*Gel consistency* (GC) is a secondary test that is aimed to further define the quality classes of varieties within the high-AC and the waxy classes. This attribute is reportedly correlated with Brabender setback viscosity but not as well as with AC (Cagampang et al., 1973). There are various versions of this method such as those that involve the use of alkali solutions (Cagampang et al., 1973; Tran, Daygon, Resurreccion, Cuevas et al., 2011) and those that involve neutral solutions (e.g., Keeratipibul, Luangsakul, & Lertsatchayarn, 2008). Varieties can be grouped into three GC classes: high (hard and very flaky texture), medium (flaky but softer rice), and low (soft and non-flaky rice). Most commonly, the gel lengths for the different GC classes are: ≤40 mm (hard), 41–60 mm (medium), and >60 mm (soft) (Graham, 2002).

*Gelatinization temperature* (GT) is typically used as an indicator of the cooking time (Cuevas, Daygon, Corpuz, Reinke et al., 2010), with three approaches being used to measure GT: (i) based on the pasting temperature from a viscosity profile (Dang & Bason, 2014); (ii) visually assessing and scoring the digestion of rice grains in an alkaline solution, aqueous potassium hydroxide (e.g., Eizenga, Ali, Bryant, Yeater  et al., 2013; Tuaño, Regalado, & Juliano, 2016); and (iii) obtaining the temperature peaks of endotherms through differential scanning calorimetry (e.g., Cuevas & Fitzgerald, 2012). It is unclear if the three methods associate with each other or if they describe other properties of the grain altogether (e.g., Shu, Shen, Bao, Wuet et al., 2006). Gelatinization temperature classes are either defined based on the temperature at which the crystalline forms of starch irreversibly melt or based on alkali spreading values. Based on temperature, rice varieties can be classified into three classes: high (>74 °C), intermediate (70–74 °C), and low (<70 °C) (Graham, 2002). Based on alkali spreading, on the other hand, there are four GT classes (in parentheses are their corresponding scores): high (2), intermediate-high (3), intermediate (4–5), and low (6–7) (Graham, 2002). However, other reports seem to indicate that there are only two classes of GT: high and low (e.g., Cuevas et al., 2010). In this classification scheme, the temperature range of the intermediate-GT class seems to be included in the low-GT class. The ambiguity in the delineations of the GT classes may pose issues to rice breeders whose targets fall at the intermediate GT levels and for people in the rice trade, as GT class may be criterion for release and market acceptance.

*Aroma* is a value-adding character to rice since it is a preferred trait by consumers. Rarely, however, do consumers describe the aroma of rice beyond the subjectivity of “with fragrance”, “no fragrance”, and “bad or unpleasant smell” (Table 1). Scientists have therefore devised means to define what aroma in rice is. Good aroma tends to be associated with pleasant aromatics found in Jasmine and Basmati rice types. Most often, the volatile compound 2-acetyl-1-pyrroline (2-AP) is found in relatively high concentrations in these aromatic rice varieties, lending a popcorn-like, cracker-like, roasted odor (Czerny, Christlbauer, Christlbauer, Fischer et al., 2008). The aroma of 2-AP is also associated with milky odor (Paraskevopoulou, Amvrosiadou, Biliaderis, & Kiosseoglou, 2014), with sweet nutty odor (Kobayashi & Nishimura, 2014), and the smell of pandan (Laksanalamai & Ilangantileke, 1993). 2-AP has a low odor threshold in water, and as such, can also be detected in varieties that are typically not flavorful (Buttery, Ling, & Juliano, 1982). The intensity of the aroma conferred by 2-AP is largely affected by both genetic background of the variety and the agronomic and postharvest conditions that the plants—and subsequently the grains—had to undergo (reviewed in Wakte, Zanan, Hinge, Khandagale et al., 2017). Although humans can smell 2-AP, the method of choice for detecting this compound on a routine basis is gas chromatography (Grimm, Bergman, Delgado, & Bryant, 2001). Aside from 2-AP, aroma in rice is defined by other volatile compounds present in the rice grain (reviewed in Anacleto et al., 2015). The presence and the varying concentrations of these other compounds make the aromas of different rice varieties distinct from each other; i.e., Basmati and Jasmine rice types. However, how these volatiles contribute to the aroma of rice is unclear; hence, defining aromatic rice is still incomplete.

*Other organoleptic properties.* The current routine indicators of cooking and eating quality address mechanical textural attributes (e.g., hardness and stickiness), cooking time, and one facet of aroma in rice. These current assays, though mostly high-throughput, have been found wanting as they do not provide a complete product profile of the organoleptic quality of a rice variety. For instance, current assays do not measure geometric sensory attributes (e.g., roughness) or a rice sample's capacity to absorb moisture; in the same token, these assays do not describe many flavors a consumer may perceive while eating rice (e.g., sweetness, mustiness, astringency). Hence, rice varieties may fall in the same quality class based on routine assays but consumers could easily distinguish them upon tasting (Champagne, Bett-Garber, Fitzgerald, Grimm et al., 2010).

Some of these gaps in rice grain quality profile can potentially be filled by measuring the intensities or detecting the presence of attributes through descriptive sensory evaluation.

The terms used in descriptive sensory evaluation are not standardized: the words used to describe the rice samples and their definitions are highly dependent on the lexicon generated prior to sensory evaluation; often, these terms reflect the relative importance of various properties over others and may be product-specific. For instance, the terms used to describe local and imported rice in West Africa include appearance attributes (e.g., discoloration, uniformity of appearance), aroma and flavor (e.g., typical odor, sweet, creamy), and mouthfeel (e.g., graininess, hardness, stickiness) (Tomlins, Manful, Larwer, & Hammond, 2005). On the other hand, textural attributes included in descriptive sensory analyses of mochi (glutinous rice cakes) focus on softness, smoothness, stickiness, and *Q*-value (Chuang & Yeh, 2006). The lexicon used by food technologists in the USDA in descriptive sensory profiling of cooked rice grains includes 13 flavor attributes, perceived through smell and taste, and 14 texture attributes (Champagne et al., 2010). The textural attributes include *mechanical properties* (e.g., hardness, stickiness) that are perceived while biting and chewing the rice, *geometric properties* that are perceived as features associated with particle size, shape, and arrangement (e.g., coarse, powdery), and properties related with perceptions of *moisture* (e.g., moisture absorption) (McKenzie, Noble, & Takami, 2006). An even more comprehensive lexicon for aroma was used by Meullenet, Griffin, Carson, Davis, Davis et al. (2001) in investigating the preferences of Asian consumers based in the United States of America. The aromatics and flavors are mostly named or described based on non-rice materials, conjuring images and descriptions that a panelist can relate to; i.e., wet cardboard, sulfur/piggy/sewer animal, burlap, hay-like, corn, and grassy/green bean.

Descriptive sensory profiling comes in different approaches. Rating scales specific for textural attributes have been developed (Szczesniak, Brandt, & Friedman, 1963). On the other hand, flavor attributes can be gauged based on a universal rating scale (Meilgaard, Civille, & Carr, 2007); alternatively, check-all-that-apply (CATA) questions can be used to describe complex aroma attributes, particularly those that are of low enough intensities that the concern becomes whether an attribute is present or not (reviewed in Varela & Ares, 2012).

As the rice market becomes increasingly complicated, with consumers becoming more demanding about the quality of the rice they purchase, it is foreseen that these attributes that are not predicted by current grain quality routine assays may become of higher importance. Sensory evaluation, however, is a low-throughput approach; therefore, it cannot be used routinely in varietal development. It is particularly useful in two arenas. First, the descriptive profiles developed through sensory evaluation provide *supplementary* information that can further differentiate rice varieties that fall within the same quality class. Second, sensory evaluation can provide a better understanding of the links from organoleptic properties (what people perceive) to intrinsic grain properties (i.e., metabolomic and structural properties), and then to the genes that code for these intrinsic grain properties. This becomes particularly important in discovering new genes of grain quality and in the development of tools that can measure these non-routine attributes in a quantitative and high-throughput manner.

## 4 Rice quality as predicted by genes

Improving yield to alleviate hunger while improving grain quality proved to be challenging in the early days of rice breeding (Zeng, Tian, Rao, Dong et al., 2017). Advances in the fields of functional genomics (e.g., The 3000 Rice Genomes Project, 2014) will eventually enable breeders to satisfy both food security and consumer preference requirements through improved definition of breeding targets using genetic data (Fitzgerald et al., 2009a). However, grain quality is defined by different combinations of different attributes (Cuevas et al., 2016; Unnevehr, 1986). Each of these attributes may be controlled by multiple sets of genes, possibly interacting with each other, leading to additive, dominant, epistatic, or pleiotropic effects (Huang, Jiang, Zheng, Wang, Wang et al., 2013) that influence the biochemical pathways that ultimately lead to functional properties. The expression of these genes may also be affected by environmental conditions. Hence, predicting grain quality based on genetics requires associating genetic information—and the possible complexities it contains—with grain quality phenotypes while taking into consideration possible interactions with the environment.

Despite these possible complications, quantitative trait loci (QTLs) associated with major indicators of grain quality have been mapped on the rice genome (reviewed in Anacleto et al., 2015). These indicators are mostly associated with starch properties. Getting the right combination of starch properties is crucial for getting the right type of rice to meet market demand. Thus, genes and QTLs that are linked with starch properties are under constant and strong selection pressure in the course of rice domestication (Singh, Singh, Rai, Sidhu et al., 2017) and rice varietal development. Chromosome 6 in rice, for instance, is reported to carry a number of major genes controlling cooking and eating quality indicators such as AC and GC (*Wx*), and GT (*SSIIa*) (reviewed in Cuevas & Fitzgerald, 2012) while minor QTLs for these traits are mapped in other chromosomes. The published *Wx* alleles have been reported to be diagnostic for eating quality and surface stickiness (Hori, Suzuki, Iijima, & Ebana, 2016), aside from AC and from GC. Mutations in these genes may lead to significant changes to starch structure, and then to variations in functional properties of starch (reviewed in Singh et al., 2017). Moreover, most of the QTLs for pasting parameters have been reported in Chromosome 6, associated mainly with the *Wx* locus (e.g., Yang, Chen, Tong, Huang et al., 2014), and in Chromosome 2 (Zhang, Zhang, Zhu, Zhao et al., 2008). The strong phenotypic associations with the *Wx* gene pose a challenge in defining grain quality (texture, in this case) because consumers are reportedly sensitive in detecting differences in textural attributes within the same AC class (Champagne et al., 2010), yet the genetic parameters that contribute to the complexities of texture are still not yet well-understood. For instance, the genetics of geometric textural attributes or of perceptions of moisture have not been fully explored, yet these attributes also contribute to the experience of eating rice and the development of consumer preferences. Hence, predicting textural quality based on genetic information requires additional in-depth exploration into the phenotype-genotype associations for textural attributes aside from hardness and stickiness (which are associated with AC). This presents an opportunity to further refine the genetic definition of textural quality within AC classes (with the same *Wx* alleles). It is not just AC, however; there is a similar challenge in predicting rice GT based on *SSIIa* haplotypes. Although there are three GT classes based on phenotype (Section 3.2), the *SSIIa* gene only classifies rice into two groups: one group with high and intermediate GT and another group with low GT (Cuevas et al., 2010; Eizenga, Ali, Bryant, Yeater et al., 2013). Current diagnostic markers for the *SSIIa* gene—or for the GT classes, for that matter—cannot differentiate between samples with high and intermediate GT; this is a challenge for varietal development programs targeting intermediate GT (Calingacion et al., 2014). There is reason to believe that there might be additional loci, other than *SSIIa*, that contribute to the intermediate GT phenotype (Cuevas et al., 2010).

Exploratory work into finding additional QTLs within the same *Wx* or *SSIIa* haplotype that could further explain cooked grain texture and cooking quality have been reported. These efforts have been made possible by the advent of high-throughput genotyping and bioinformatics capacities. For instance, Yang et al. (2014) and Li, Bao, Corke, & Sun, 2017 have used the *Wx* and *SSIIa* SNPs as covariates in an association mapping approach that takes the gross-level population structure and kinship (Q + K) into account. Results from their studies indicate that variability within the same GT class is associated with several SSR markers and other starch synthesis-related genes such as *Wx*, *SSIIc*, *SBE1*, *SSIIb*, and *ISA3*. Meanwhile, *SBE1*, *SSIIc*, *GBSSII*, *SSIIa*, and *SSIIIb*, all starch synthesis-related genes, were associated with variation within the same AC class. Additionally, the very low-AC genotypes in a Guanxi germplasm collection were associated with a novel gene, *LAC6*, which most likely codes for a retrotransposon protein (Yang, Nong, Xia, Zhang et al., 2017).

Grain weight is crucial in determining the yield in rice; hence, it is an attribute that breeders have been targeting to achieve food security and self-sufficiency goals. Grain weight depends on two factors: grain dimension and grain filling. *Grain dimension*, aside from being important for yield, is also an important intrinsic quality cue (as discussed in Section 2.1). Huang et al. (2013) reported that grain dimension is associated with 13 cloned genes and over 400 QTLs; 17 of these QTLs have been mapped in different chromosomes. More are being reported as the technological approaches required to associate genotype with phenotype are improving. Some of these genes and QTLs, plus newly identified ones, have been associated with multiple biochemical signaling pathways (e.g., Yuan, Fan, Huang, Zhan et al., 2017), which illustrate the complexity of regulation for this attribute. However, many of these studies utilized biparental crosses to find these QTLs (e.g., Takano-Kai, Jiang, Kubo, Sweeney et al., 2009; Wan, Weng, Zhai, Wang et al., 2008); hence, these QTLs might be specific for these particular populations and may not be applicable for molecular marker development of screening tools for breeding programs using different parent materials and with specific grain size targets. Moreover, for many of these QTLs, the biochemical pathways that they control are not yet elucidated; it remains to be seen if more of these QTLs will be developed into tools that can predict quality in rice. Factors that make the genetics of grain size regulation difficult to isolate include environmental interactions that affect gene expression and constant interactions between the endosperm and the integuments of the developing seed (reviewed in Zuo & Li, 2014). *Grain filling*, on the other hand, has been reported to involve 33 major enzymes in the developing grain linked with carbohydrate metabolism and starch synthesis (reviewed in Chen, Yan, Wang, Gao et al., 2017). This indicates that the genetics of grain filling, an activity that contributes to chalkiness, HR, and grain appearance (Section 3.1), is complex. Some progress has been achieved in determining the gene(s) that lead to chalkiness and two QTLs have been identified for HR (Wang et al., 2017). The effect of environmental conditions on the functionality of genes associated with parameters such as HR (Li et al., 2018), further demonstrate the complexity of the grain filling process. Because the genetics of grain filling rate is not well understood, it is typically skipped when breeders develop trait targets. For instance, gene expression experiments suggest that there might be unknown pathways that regulate the expression of starch-synthesizing genes in the endosperm; the known and the unknown pathways that affect grain filling rates are involved mainly in amylopectin synthesis (Inukai, 2017). This is expected because glutinous rice varieties, those that have the null *wx* allele, do not have amylose (Table 5) but have amylopectin. Amylopectin synthesis involves various enzymes such as soluble starch synthases, branching enzymes, and debranching enzymes (reviewed in Tetlow, Morell, & Emes, 2004) aside from those mentioned that are closely associated with GT. This indicates that there are potentially more genes involved in grain filling that could be used as tools, individually or in combination, for predicting this quality attribute. Perhaps, further advancement in genotype-phenotype association techniques will lead to a deeper understanding of the pathways involved in grain filling and the numerous genetic factors that lead to certain grain weight phenotypes.

In contrast to the genetics of grain weight, it appears that there is a much more comprehensive understanding about the genetics and the biochemistry of aroma in rice. One recessive gene (referred to as *Fgr*, as *BADH2*, or as *Os2AP*) mapped on Chromosome 8 codes for the betaine aldehyde dehydrogenase (BADH) enzyme; which, if non-functional, has been associated with the biosynthesis of the volatile compound 2-AP (Section 3.2) (Kovach, Calingacion, Fitzgerald, & McCouch, 2009). Aside from the non-functionality of *BADH2*, the overexpression of the gene *OsP5CS* (mapped on Chromosome 5), coding for the Δ^1^-pyrroline-5-carboxylate synthase (P5CS), is also correlated with the 2-AP content in rice because it is associated with the enhanced synthesis of proline, a 2-AP precursor (reviewed in Wakte et al., 2017). Ornithine and glutamate are alternate starting materials for 2-AP synthesis via the pathway involving P5CS as well (Huang, Teng, Chang, Chuang et al., 2008). Aside from 2-AP synthesis, P5CS is associated with drought and salinity tolerance (Hien, Jacobs, Angenon, Hermans et al., 2003). Overlapping metabolite pathways to 2-AP accumulation probably contribute to reasons why aromatic rice varieties tend to thrive in areas with unfavorable growth conditions (reviewed in Mo et al., 2016. Aside from the gene and environmental stress interactions, reports have shown that mineral elements have effects on the expression of the aromatic phenotype (Mo, Huang, Xiao, Ashraf et al., 2016).

Other quality attributes, particularly geometric and moisture attributes (Section 3.2), and volatile compounds in rice that contribute to fragrance have not been associated with genes or with QTLs. Given the high-throughput nature of current genotyping and association methods, the limitations for developing molecular markers for these attributes are (i) the ability to quantitatively and objectively phenotype those attributes (i.e., not be limited to ratings based on sensory perceptions), and (ii) the capacity to identify and to link unknown volatile compounds with sensory perception.

However, even if the genetic factors influencing the routine and the additional grain quality attributes are elucidated, the genetic information remains a predictive tool indicating the quality of a rice sample. This is because genetics indicates predisposition towards a certain characteristic. The interplay between genetics and environment can greatly influence gene expression, potentially leading towards phenotypes that are different from what the genes code.

## 5 Conclusions, implications and future perspectives

The rice sector currently lacks consensus on how “rice quality” should be defined and measured, despite its crucial role in VC upgrading. An inter-disciplinary review of consumer, farmer and industry perceptions, food science and technology, and genetics literature reveals that rice quality is context-specific and consumers are heterogeneous with respect to their perceived quality differentiation among regions, countries, cities, and urbanization levels. However, within a given context, consumers from diverse SECs seem to remarkably agree on how “poor”, “good”, or “premium” quality rice is defined, although these quality classes are not perceived as being distinct, but rather as a gradient. In SEA, premium rice quality features nutritional benefits, softness and aroma, while in SA the emphasis is more on the physical appearance of the uncooked grains (uniformity in size and shape, whiteness, slenderness) and the satiety the cooked product delivers, characteristics that SEA consumers take for granted for any rice product superior to “poor” quality rice. These trends are found to be consistent with farmer and industry perceptions, suggesting that rice VCs successfully transmit consumer perceptions to rice VC actors in SEA and SA. These regional and national specificities have important implications for the division of labor between regional and national breeding programs. Regional programs can incorporate “regional traits” into germplasm to be transferred to national programs, which can further develop regionally targeted germplasm into varieties tailored to specific national and local market segments. Rice VC actors can further reinforce rice quality through labeling, branding, and packaging, especially in Thailand, Cambodia, South India, and Bangladesh where consumers are used to purchase packaged rice.

Because rice is traded internationally, there is a need to standardize definitions of rice quality that can be used globally. However, food technologists have yet to reach unanimity as to how rice quality should be quantitatively measured and categorized particularly with respect to grain size and shape, chalkiness, HR, AC, and GT. There is often a misalignment between food scientists and VC actors on quality classes, which may lead to potential issues in rice trading and prevent rice breeders from setting relevant breeding trait targets. Although demand for aroma is increasing in SEA and SA, defining aromatic rice is still incomplete as aroma is determined by the presence and the varying concentrations of 2-AP and other volatile compounds in the rice grain. As the current routine indicators of cooking and eating quality do not provide a complete product profile of the organoleptic quality of a rice variety, descriptive profiles need to be elicited through a sensory evaluation panel to further differentiate rice varieties that fall within the same quality class.

Because each of the quality attributes of rice may be controlled by multiple sets of genes and the expression of these genes may be affected by gene interactions, crop management, and environmental conditions, predicting grain quality based on genetics requires associating genetic information with grain quality phenotypes in different environments. Genetics research has focused on starch properties as the latter is crucial for tailoring rice to the specificity of consumer preferences in SEA versus SA. However, the genetic parameters that contribute to the complexities of texture and grain filling rate remain poorly understood, hampering the development of precise breeding trait targets. In contrast, the the interactions among genetics, biochemistry, and environmental factors leading to aroma phenotypes are better understood. Aroma in rice tends to express itself better under environmental stress conditions, which explains why these varieties are often found in unfavorable rice growing environments.

Our review identified several challenges in defining rice quality from consumers to genetics and the need for an international body, such as IRRI, to standardize international quality indicators for rice. The more rice VCs become demand-driven with modernization of retail and globalization of trade, the more urgent a uniform definition of rice quality becomes. This definition is important for consumers to match their quality expectations with their preferences; for VC actors to match consumers’ expectations with supply and pricing; for rice breeders to set relevant breeding trait targets that correspond to regional preferences and local market segments; and finally for policy-makers to set relevant targets for investment in agricultural research, infrastructure, and value chain upgrading to increase regional and national food and nutrition security.

## Funding

This work was supported by the Bill & Melinda Gates Foundation, Seattle, WA, USA [Grants no. OPP1076488 and OPP1194925]; and the CGIAR Research Program on Rice.

## Appendix A Supplementary data

APPLICATION; MMC1:TIFS v6.0.docx(56-58) APPLICATION; MMC1:TIFS v6.0.docx(56-58)
