# Supplementary material for: Rice quality: How is it defined by consumers, industry, food scientists, and geneticists?
Source: Trends Food Sci Technol. 2019 Oct;92:122–37. doi: 10.1016/j.tifs.2019.07.039 (PMC6876681; doi:10.1016/j.tifs.2019.07.039)
Supplement: APPLICATION; MMC1:TIFS v6.0.docx(56-58) [file mmc1.docx]

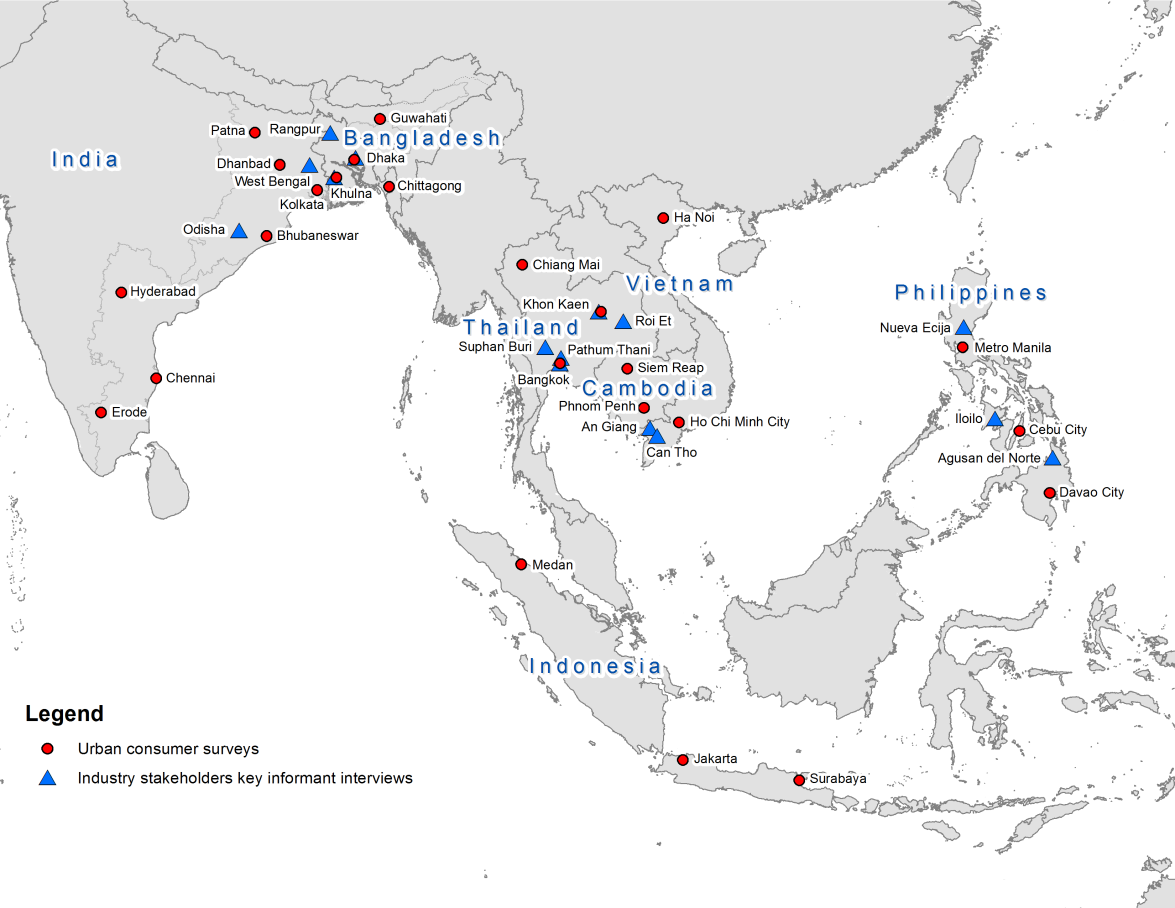


**S1**

Urban consumer survey sites and locations of FGDs with farmers and key informant interviews with value chain actors conducted in 2013–2014.

**S2**

Sample sizes of urban consumer surveys, FGDs with farmers and key informant interviews with value chain actors conducted in 2013–2014.

|  | Urban SEA | Thailand | Philippines | Indonesia | Vietnam | Cambodia | Urban SA | East India | South India | Bangladesh |
| --- | --- | --- | --- | --- | --- | --- | --- | --- | --- | --- |
| Urban consumers | 2,150 | 500 | 500 | 500 | 300 | 350 | 1,923 | 803 | 619 | 501 |
| FGDs with farmers  (# of groups) | 15 | 3 | 6 | *na* | 6 | *na* | 15 | 7 | *na* | 8 |
| Key informant interviews with other value chain actors | 50 | 14 | 23 | *na* | 13 | 40 | 44 | 22 | *na* | 22 |

*Notes:* FGDs with farmers and key informant interviews with value chain actors were not conducted in Cambodia, Indonesia and South India. The sample sizes of rural consumer surveys in East India and Bangladesh are 497 and 599, respectively.
